# Using Participatory Learning & Action (PLA) research techniques for inter-stakeholder dialogue in primary healthcare: an analysis of stakeholders’ experiences

**DOI:** 10.1186/s40900-017-0077-8

**Published:** 2017-12-06

**Authors:** T. de Brún, M. O’Reilly - de Brún, E. Van Weel-Baumgarten, N. Burns, C. Dowrick, C. Lionis, C. O’Donnell, F. S. Mair, M. Papadakaki, A. Saridaki, W. Spiegel, C. Van Weel, M. Van den Muijsenbergh, A. MacFarlane

**Affiliations:** 1 Centre for Participatory Strategies (CPS), Galway, Ireland; 2 Discipline of General Practice, School of Medicine, National University of Ireland, Galway, Ireland; 3 Department of Primary and Community Care, Radboud University Medical Center Nijmegen, Nijmegen, The Netherlands; 4 Lancaster Medical School, Faculty of Health and Medicine, Lancaster University, Lancaster, LA1 4YW UK; 5 Department of Psychological Sciences, B121 Waterhouse Buildings University of Liverpool, Liverpool, UK; 6 University of Crete, Faculty of Medicine, Clinic of Social and Family Medicine, Heraklion, Greece; 7 General Practice & Primary Care, Institute of Health and Wellbeing, College of Medical Veterinary and Life Sciences, University of Glasgow, Glasgow, UK; 8 Technological Educational Institute of Crete, School of Health and Social Welfare, Department of Social Work, Heraklion, Greece; 9 Centre for Public Health, Medical University of Vienna, Kinderspitalgasse 15/1st floor, A-1090, Vienna, Austria; 10 Pharos, centre of expertise on health disparities, Utrecht, The Netherlands; 11 Graduate Entry Medical School, University of Limerick, Limerick, Ireland

## Abstract

**Plain English summary:**

It is important for health care workers to know the needs and expectations of their patients. Therefore, service users have to be involved in research. To achieve a meaningful dialogue between service users, healthcare workers and researchers, participatory methods are needed. This paper describes how the application of a specific participatory methodology, Participatory Learning and Action (PLA) can lead to such a meaningful dialogue. In PLA all stakeholders are regarded as equal partners and collaborators in research.

During 2011–2015, a European project called RESTORE used PLA in Austria, Greece, Ireland, The Netherlands and the UK to investigate how communication between primary health care workers and their migrant patients could be improved.

Seventy eight migrants, interpreters, doctors, nurses and other key stakeholders (see Table 2) participated in 62 PLA sessions. These dialogues (involving discussions, activities, PLA techniques and evaluations) were generally 2–3 h long and were recorded and analysed by the researchers.

Participants reported many positive experiences about their dialogues with other stakeholders. There was a positive, trusting atmosphere in which all stakeholders could express their views despite differences in social power. This made for better understanding within and across stakeholder groups. For instance a doctor changed her view on the use of interpreters after a migrant explained why this was important. Negative experiences were rare: some doctors and healthcare workers thought the PLA sessions took a lot of time; and despite the good dialogue, there was disappointment that very few migrants used the new interpreting service.

**Abstract:**

**Background**

In order to be effective, primary healthcare must understand the health needs, values and expectations of the population it serves. Recent research has shown that the involvement of service users and other stakeholders and gathering information on their perspectives can contribute positively to many aspects of primary healthcare. Participatory methodologies have the potential to support engagement and dialogue between stakeholders from academic, migrant community and health service settings. This paper focuses on a specific participatory research methodology, Participatory Learning and Action (PLA) in which all stakeholders are regarded as equal partners and collaborators in research.

Our research question for this paper was: "Does the application of PLA lead to meaningful engagement of all stakeholders, and if so, what elements contribute to a positive and productive inter-stakeholder dialogue?".

**Methods**

We explored the use of PLA in RESTORE, a European FP7-funded project, during 2011–2015 in 5 countries: Austria, Greece, Ireland, the Netherlands and the UK. The objective of RESTORE was to investigate and support the implementation of guidelines and training initiatives (G/TIs) to enhance communication in cross-cultural primary care consultations with migrants.

Seventy eight stakeholders (migrants, interpreters, doctors, nurses and others – see Table 2) participated in a total of 62 PLA sessions (discussions, activities, evaluations) of approximately 2–3 h’ duration across the five sites. During the fieldwork, qualitative data were generated about stakeholders’ experiences of engagement in this dialogue, by means of various methods including participatory evaluations, researchers’ fieldwork reports and researcher interviews. These were analysed following the principles of thematic analysis.

**Results**

Stakeholders involved in PLA inter-stakeholder dialogues reported a wide range of positive experiences of engagement, and very few negative experiences. A positive atmosphere during early research sessions helped to create a sense of safety and trust. This enabled stakeholders from very different backgrounds, with different social status and power, to offer their perspectives in a way that led to enhanced learning in the group – they learned with and from each other. This fostered shifts in understanding – for example, a doctor changed her view on interpreted consultations because of the input of the migrant service-users.

**Conclusion**

PLA successfully promoted stakeholder involvement in meaningful and productive inter-stakeholder dialogues. This makes it an attractive approach to enhance the further development of health research partnerships to advance primary healthcare.

## Background

Concerns about increasing health care expenditure with diminished health-related returns on investment are the driving factor behind many health reforms. In parallel with this, there is a paradigm shift towards person-centred care that is responsive to individuals, groups and communities [1, 2]. Responsive, effective primary healthcare systems are an important element of this development [3, 4]. In order to be effective, primary healthcare must be informed by an accurate understanding of the diverse health needs, values and expectations of the populations they serve-, and how these are affected by the social and cultural determinants of health [3–6]. This requires an exchange and information gathering process between primary healthcare professionals and patients/service-users in individual encounters, as well as in research and service planning. This paper relates to patient and public engagement in primary healthcare research partnerships [7–12] with a and our specific focus is on migrant health. There are increasing imperatives for involving patients and members of the public in health research. There is international support for Public and Patient Involvement (PPI) in the form of legislation and policy directives [13] and it is increasingly advocated by health funding agencies and Government health departments [7–9, 11, 12, 14].

The first international evidence about the impacts of PPI at all stages of research from design to dissemination is well-documented in the PIRICOM study, a recent systematic review of 66 PPI studies. PIRICOM indicated that the involvement of stakeholders (see Table 1) can make positive practical contributions to many aspects of primary healthcare research, for example, fostering greater honesty in the flow of information during data-generation and a broader interpretation of data during data-analysis [15]. Pearson et al. also note the positive value of stakeholders’ insights regarding, for example, service-design, quality and safety [16].

**Table 1:** Definitions and descriptions of key terms

Researcher/catalyst
Researchers who adopt a PLA approach, techniques and mode of engagement act as catalysts – their primary role and responsibility is to elicit diverse stakeholders’ perspectives and facilitate collaborative inter-stakeholder dialogue/action. The researcher/catalyst facilitates, rather than controls, the direction that stakeholder’s perspectives provide to the research process [60].
PLA ‘mode of engagement’
A PLA ‘mode of engagement’ is the essential attitudinal disposition a researcher/catalyst adopts to promote participation, learning and positive action by and with diverse stakeholder groups; the researcher/catalyst listens, enables, supports stakeholder/inter-stakeholder dialogues, which are ideally reciprocal, mutually respectful, co-operative and productive [19, 31]. Where necessary and appropriate, researchers may act as knowledge-brokers, sharing perspectives and insights that emerge from one stakeholder group with the next, thus ‘brokering’ an educative inter-stakeholder dialogue [19, 20].
PLA research methods and techniques
The broad range of qualitative, participatory activities typical of PLA research which combine the verbal, visual and tangible. Verbal activity includes focus groups, interviews, dialogues, debate and negotiation, story-telling, oral histories, role-play and drama. These are usually combined with visual and tangible activity – generating physical maps, charts, diagrams (e.g., Commentary Charts, Direct Ranking, Seasonal Calendars). Stakeholders’ priorities and perspectives guide this participatory engagement process [19, 20. 31, 55, 60].
Meaningful engagement
We draw from the work of Cornwall and Jewkes, [18] Gaventa [21] and Chambers [22] in defining ‘meaningful engagement’ as an experience of partnership in research that is collegial, inclusive and active for participants, reduces asymmetries of power and enables participants’ authentic perspectives to emerge clearly in research outcomes. A PLA mode of engagement, research methods and techniques are intended to support and facilitate meaningful engagement in inter-stakeholder dialogues.
Stakeholder
Drawing from McMaster Health Forum (2015) we describe a stakeholder as an individual, group or organisation that has an interest in the organisation and delivery of healthcare and will have an interest in the content or outcome of a guideline.
Inter-stakeholder dialogue
Drawing from the work of McMaster [81] regarding ‘stakeholder dialogue’, and Pronk [82] and Ashraf [83] regarding ‘multi-stakeholder dialogue’ we describe inter-stakeholder dialogue as a multi-directional dialogue that occurs between/among diverse stakeholders. Diverse stakeholders may coalesce into a single group, or be dispersed across several groups. Inter-stakeholder dialogues focus on a shared goal (e.g. a health issue or problem all stakeholders have a vested interest in addressing/solving). By learning from each other’s perspectives, stakeholders may develop unique understandings or uncover insights related to the issue or problem at hand, generating creative solutions regarding key implementation considerations. This can only come about when relevant stakeholders who are involved in, or affected by decisions related to the issue, work through it together, charting an agreed course of action towards their shared goal.
Transformative moments
During PLA research, transformative moments can occur when stakeholders (working in a group or across several groups) face a seemingly intractable problem and, through dialogue, some stakeholders make a small leap of generosity towards others, thereby resolving the impasse [65, 84].

However, as early as 2002, Beresford warned that increasing pressures for stakeholder involvement from a wide range of sources could lead to it being seen as a ‘must-do, virtuous activity’ which can lead to a ‘tick-box’ tokenistic approach, simply going through the motions, but failing to go beyond a *consumerist approach.* He emphasises the value of a *democratic approach* which involves stakeholders in powerful decision-making roles in research to enable them to improve the quality of their lives [17]. The current literature on PPI in research indicates that involvement spans a continuum from inclusion of stakeholders as passive subjects of a study (tokenistic involvement) to the inclusion of stakeholders as active participants, collaborators and contributors to many aspects of research activity (meaningful engagement – see Table 1) [18–26]. For some patient groups, active participation is even more challenging than for others. For instance, migrants, the focus of RESTORE, are less often included, let alone actively participate in research, due to communication barriers and unfavourable socio-economic living conditions [27].Domecq, Prutsky et al. [28] reviewed 142 studies that described a spectrum of engagement and concluded that, while engagement seems feasible in most cases, both service-users and researchers had overarching concerns about tokenism. Their review is consistent with previous reviews in the field of PPI [15, 26, 29, 30] and they conclude that there is limited knowledge about particular methods for enacting meaningful engagement and that research dedicated to identifying the best methods for this is lacking and clearly needed [28]. Similarly, following Jagosh et al. [6] who emphasise the importance of *partnerships for health research*, it is clear we need to know more about effective methods for developing research partnership*s* that can facilitate meaningful dialogue between stakeholders from academic, community and health service settings. These need to be supported by a relational environment of trust [31] and mutual respect, generating ‘safe spaces’ where stakeholders feel secure and empowered to actively participate. The challenge is not to subsume differences of opinion and knowledge, but to enable stakeholders to deal positively with differences and to seek mutually acceptable outcomes. At its best, then, inter-stakeholder dialogue (see Table 1) has the potential to empower diverse stakeholders to contribute their unique insights to the co-generation of knowledge in research processes. This challenging task of engaging stakeholders (who may hold diametrically opposing views) in productive inter-stakeholder dialogue requires a research approach, methodology and techniques suited to the task. To address these problems and challenges of PPI, it is valuable to draw on a ‘bottom-up’ participatory research methodology that is inherently dialogic in nature [30, 32–34].

There is a range of participatory methodologies including, among others, Participatory Research (PR) [5, 18, 35, 36], Participatory Action Research (PAR) [37, 38], Community Based Participatory Research (CBPR) [39–45], Participatory Rural Appraisal (PRA) [22, 46, 47] and Participatory Learning & Action (PLA) [19, 20, 32, 48]. All share a democratic ethos, are strongly committed to meaningful engagement by stakeholders, and promote research partnerships that strengthen relations between academy and community [6, 49–52]. Participatory approaches emphasise the need for stakeholders’ active engagement across the full range of research activities [50, 53].

This paper focuses on PLA because recent research studies in primary healthcare indicate the scope for meaningful research partnerships with migrants [19, 20, 32, 54, 55] which can be realised by PLA, and warrant further investigation.

PLA is a form of action research, rooted in interpretive and emancipatory paradigms [56] and influenced by the work of Robert Chambers [47, 48]. It is a practical approach to research with diverse stakeholders where different world-views pertain and asymmetries of power may exist and need to be balanced [22, 46–48, 57]. An important principle of PLA is the reversal from a perception of stakeholders (see Table 1) solely as beneficiaries to a perception that stakeholders are also partners and collaborators in research [32]. Researchers use a PLA ‘mode of engagement’, and act as catalysts to support an inclusive atmosphere and positive tone with and among stakeholders. A key aim is to create ‘safe space’ and trusting relationships [58] in which stakeholders can feel sufficiently secure to risk sharing diverse perspectives and opinions. Researchers actively encourage stakeholders to recognise that they are ‘experts’ in their own right, possessing unique *implicit* knowledge of their lives and conditions [19, 22, 34].They bring their valuable knowledge to the ‘stakeholder table’, where, by participating in a range of PLA techniques, they engage in iterative cycles of discussion and dialogue which makes their knowledge *explicit* and therefore available to the research. The inclusive, user-friendly and democratic nature of PLA techniques also encourages stakeholders to co-operatively assess, generate and analyse data, to create workable solutions to problems that may arise during research, and to plan for change and/or implementation [59, 60]. In practical terms, stakeholders achieve this through the generation of maps, charts, diagrams (visual and tangible data) while engaging in one or other form of discussion/dialogue, e.g., focus groups, interviews, story-telling, drama, role-plays (verbal data). The production of maps and charts is not the primary goal – the knowledge-exchange, knowledge-enhancing process that goes on among stakeholders as they work together on these tasks is key to the inter-stakeholder dialogue and the research outcomes [61]. As inter-stakeholder dialogues develop, the knowledge-exchange, knowledge-enhancement process can lead to learning the new or the unexpected and stakeholders may experience shifts and changes in their understanding. This occurs in a relational environment that tends to be characterised by *co-operation* rather than *competition*. While the use of PLA in primary healthcare research is growing and migrants’ experiences of the methods have been reported [19], there has been no detailed analysis to date of the use of PLA methods for *inter-stakeholder dialogues with migrants* in health research. The sharp rise in migration (especially of refugees) to the European Union in recent years lends an urgency to addressing this gap in knowledge [62].

In this paper, our objective is to address this knowledge gap by exploring the use of a Participatory Learning & Action (PLA) research methodology for inter-stakeholder dialogue in a recent European primary healthcare implementation project – the RESTORE project. We use empirical evidence of stakeholders’ experiences of, and researchers’ reflections on this type of dialogue to develop and present an analysis intended to enhance our understanding of what makes for *positive* and *productive* inter-stakeholder dialogue. Our main research question to be answered in this paper was: "Does the application of PLA lead to meaningful engagement of all stakeholders, and if so, what elements contribute to a positive and productive inter-stakeholder dialogue?".

In Table 1 we present definitions and descriptions of key terms used throughout this paper.

## Methods

### Study setting and design – The RESTORE project

RESTORE (**RE**search into implementation **ST**rategies to support patients of different **OR**igins and language background in a variety of **E**uropean primary care settings) was an EU FP7-funded project running from 2011 to 2015. The objective of RESTORE was to investigate and support the implementation of guidelines and training initiatives to enhance communication in cross-cultural primary care consultations. This was a qualitative, comparative case-study [63] informed by Normalisation Process Theory (NPT) [64]and PLA.

RESTOREinvolved diverse stakeholders across five primary care settings with diverse primary healthcare systems: Austria, England, Greece, Ireland and The Netherlands [65].A sixth research team in Scotland focussed on policy-related implications of the study. The choice of countries matched the academic teams who developed the original FP7 proposal and intentionally included countries with diverse primary health care systems [54]. Ethical approval was granted by the respective national committees.

**Fig. 1:**
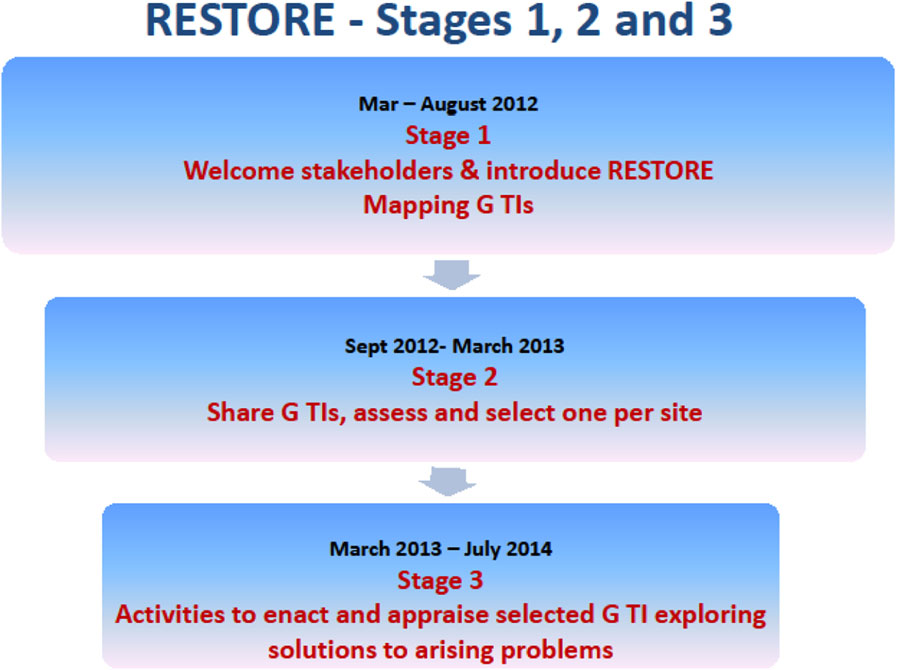
The three stages of RESTORE

RESTORE was designed as part of a series of participatory research studies with migrant community involvement exploring communication in cross-cultural consultations [20, 66]. The design and governance of RESTORE was led by an academic consortium but, following the principles of PLA, the research process (recruitment, fieldwork and data analysis) was inclusive of migrants and other stakeholders with significant ownership of decision-making about methods, pace of work and the implementation work in hand. There were three stages to RESTORE (see Fig. 1). A detailed description of the study protocol [67] and the rationale for combining NPT [64] and PLA [19, 20, 32] is published elsewhere. Details of the implementation process and its outcomes are also available [59, 68, 69], and these analyses highlight the central role that the PLA methodology had on both process and outcome.

Therefore, our focus in this paper is on our use of PLA as a methodology and range of techniques used to engage stakeholders in inter-stakeholder dialogue as they undertook parallel, but distinctive, implementation journeys during stage 2 and 3 of RESTORE.

RESTORE was the *overall setting* in which we explored the use of PLA for inter-stakeholder dialogue.

### Sampling and recruitment of stakeholders

The primary care setting in each country determined the make-up of the stakeholder groups (see Table 2) we were able to access and engage in inter-stakeholder dialogues in each country.

**Table 2:** Stakeholders’ socio-demographic characteristics

Gender	Austria	England	Greece	Ireland	Netherlands
Male	6	2	6	3	8
Female	9	7	10	8	19
Age group					
18–30	3	2	3	0	2
31–55	9	7	11	11	20
56 plus	3	0	2	0	5
Country of origin					
Chile	–	–	–	1	–
Congo	–	–	–	1	–
Ireland	–	–	–	3	–
Nigeria	–	–	–	1	–
Poland	–	–	–	1	–
Portugal	–	–	–	1	–
Russia	–	–	–	1	–
Netherlands	–	–	1	1	22
Morocco	–	–	–	–	1
Indonesia	–	–	–	–	3
Philippines	2	–	–	–	1
Greece	–	–	13	–	–
Syria	–	1	1	–	–
Albania	–	–	1	–	–
UK	–	6	–	–	–
Pakistan	–	1	–	–	–
Austria	7	–	–	–	–
Croatia	2	–	–	–	–
Turkey	2	–	–	–	–
Ghana	1	–	–	–	–
Benin	1	–	–	–	–
Undefined	–	1	–	–	–
Stakeholder group					
Migrant community	8	7	2	8	8
Primary care doctors	5	1	4	1	8
Primary care nurses	1	0	5	0	2
Primary care admin/management staff	1	0	1	1	6
Interpreting community	0	1	0	5	0
Health service planning and/or policy personnel	5	1	5	1	4

In this paper we focus on RESTORE Stakeholders (SH):

Migrant service user (MSU).

Interpreter (Int).

General Practitioner / Family Physician (GP).

Practice Nurse (PN) (in Austria referred to as Doctor’s surgery assistant).

Practice Manager (PM).

Researcher (Res).

Purposeful and network sampling [70] was used to identify and recruit 78 stakeholder representatives in primary care settings across the five sites for Stages 2 and 3 research (see Table 2 for details). Stakeholders included migrant service-users, general practitioners / family physicians (GPs), primary care nurses, practice managers and administrative staff, interpreters and cultural mediators, service planners and policy-makers. They participated in a total of 62 PLA sessions, spread over a period of 15 to 19 months, the majority of which were 2–3 h in duration. The extensive nature of participation in terms of the number of months anticipated for fieldwork and the intention to have 2–3 h PLA sessions at regular intervals was discussed explicitly during recruitment.

RESTORE researchers were trained in a PLA mode of engagement and series of techniques to facilitate and support inter-stakeholder dialogues. The Irish team (MacFarlane, O’Reilly-de Brun and de Brun) had previous experience with PLA.

The PLA training programme was provided by two members of the RESTORE consortium (1st and 2nd author) who are PLA practitioner/trainers, with over 25 years’ international experience in PLA research and training in diverse cultural and social settings [19, 20, 32, 67, 71–73].

The extensive nature of the time commitment for stakeholders and researchers was known at the outset and was explicitly discussed during recruitment and consortium meetings respectively. This issue was re-visited during consortium meetings which were held every 6 months in order to be responsive to emergent challenges. However because of the participatory nature of PLA it was not possible to predict precisely at the start how much time would be needed.

### PLA techniques used in RESTORE

**Fig. 2:**
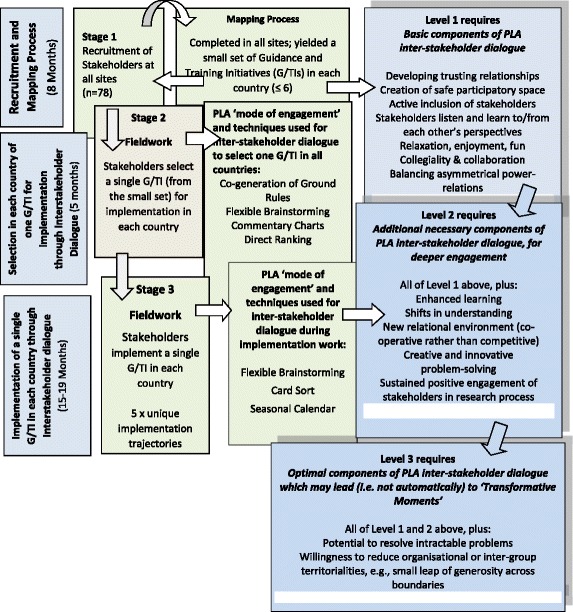
Flowchart

The uniqueness of the location, context and differing primary care systems, coupled with the Guideline or Training Initiative chosen, meant that each country followed its own unique implementation ‘journey’, or trajectory. These specifics meant that different combinations of PLA techniques were used to suit individual local settings. It is not possible to do justice to this complexity in this paper, but to gain a sense of what PLA ‘looks like’ in action during an inter-stakeholder dialogue, please see Table 3 and Fig. 2, which provide brief descriptions of the PLA techniques used in stage 2 and 3 of RESTORE fieldwork, and Table 4 which presents a practical description of a PLA session that took place at the Irish site.

**Table 3:** Stage 2 and 3 Fieldwork: Description of PLA techniques

Stage 2 fieldwork: Description of PLA techniques
Co-generated Ground Rules A democratic decision-making group activity that usually occurs at the outset of a PLA research cycle or process.
*Aim & Rationale:* Generates a set of agreed rules for stakeholders’ and researchers’ co-participation, interaction, dialogue, and joint activity during a PLA research cycle or process.
Encourages active inclusion and early co-ownership by stakeholders of PLA research activities, promoting empowerment.
Helps to balance asymmetrical power relations in and between stakeholder groups where these may exist.
Commentary Charts An interactive, highly-visual charting or diagramming technique promoting knowledge-exchange and knowledge-enhancement during stakeholder/inter-stakeholder dialogues.
*Aim & Rationale:* Enables stakeholders to learn from each other’s differential knowledge, expertise and perspectives, broadening horizons and advancing stakeholder dialogue.
Generates visual ‘data displays’ of stakeholders’ perspectives and knowledge about the issue being explored, including ‘positive’ and ‘negative’ aspects of each as described from diverse stakeholders’ perspectives, in their own words.
Commentary Charts are useful ‘data displays’ and aide-memoires that all stakeholders can review prior to engaging in Direct Ranking.
Direct Ranking A democratic ranking/prioritization technique.
*Aim & Rationale:* Enables stakeholders to democratically and transparently prioritise a set of items.
Generates visual outcome of stakeholders’ democratic decision.
Advances inter-stakeholder dialogue towards the task in hand.
Stage 3 fieldwork: Description of PLA techniques
Flexible Brainstorming An interactive knowledge-generation and knowledge-exchange technique. *Aim & Rationale:* Enables stakeholders to rapidly generate and share data in a co-operative manner, using visual materials which are flexible arranged and re-arranged and often brought forward into subsequent techniques.
Card Sort A categorization technique.
*Aim & Rationale:* Enables stakeholders to generate analytical categories that are meaningful to them and to arrange data within these categories. Particularly useful for stakeholder’s assessment and co-analysis of data.
Seasonal Calendar A grid-based diagram involving co-operative stakeholder/inter-stakeholder dialogue, action-planning and decision-making – the diagram includes a stakeholder-informed timeframe and set of identified actions necessary for an implementation process.
*Aim & Rationale:* Enables stakeholders to dialogue, assess and negotiate assignment of responsibilities (activities, tasks) as they co-plan their work.
Seasonal Calendars are useful as a ‘running record’ of stakeholders’ fine-tuning of action-planning, and a record of emerging outcomes of implementation/action over time.
Diagram can be computerized, readily shared among dispersed stakeholder groups, and populated with revisions, additions, deletions and updates.
Speed Evaluation (SE) A brief verbal (digitally recorded or written) evaluation, usually conducted at the close of a PLA process or session. Stakeholders respond to open-ended questions, in a rapid, interactive and spontaneous way.
*Aim & Rationale:* Provides stakeholders with an opportunity to describe experiences in their own words, affirming positives and suggesting areas for improvement.
Allows researchers to ‘take the temperature’ of the group, to build on positives, and, where possible, to plan suggested improvements for forthcoming PLA sessions.
Coming at the close of a PLA session, speed evaluations can be as short as ten minutes, are not unduly demanding, yet yield valuable formative evaluation data.
Participatory Evaluation (PE) A form of in-depth collaborative evaluation (formative or summative) which is based on a combination of *etic* and *emic* criteria. Etic criteria are identified in advance by researchers, whose experience enables them to suggest valuable ‘outsider’ criteria. Emic criteria emerge from shared ‘insider’ experiences, are identified by participants themselves and enable them to suggest valuable ‘insider’ criteria. The final democratically-agreed set of emic and etic criteria forms the evaluation parameters.
*Aim & Rationale* Provides stakeholders with an opportunity to suggest evaluation criteria which are important and meaningful to them.
Stakeholders’ emic criteria are capable of yielding evaluation data about the affective dimension of their experience, which often drives behavior but might otherwise remain ‘invisible’ and ‘unheard’.
Researchers often note that emic criteria contribute to an evaluation in ways they could not have anticipated or planned.

**Table 4:** Description of inter-stakeholder dialogue, Stage 2 fieldwork - Direct Ranking PLA technique

Description of inter-stakeholder dialogue, Stage 2 fieldwork: Direct Ranking PLA technique
On the evening of 6th February 2013, eleven people who are stakeholders in the RESTORE research project about migrant health gather for the seventh time in a meeting room in the National University of Ireland, Galway. Having come from various workplaces, they are greeted with culturally-appropriate refreshments. Aged between 31 and 55, eight are female, three male. Of these, eight represent migrant communities from six different countries and cultures, and five of them have experience in community interpreting. Also present are a policy planner, a practice manager and a doctor. These stakeholders make up an ‘inter-stakeholder group’ as they represent various and diverse backgrounds and fields of stakeholder expertise; they all have a vested interest in participating in the research and all have unique knowledge to contribute. All are fluent in English which is the conversation language. Two researchers from the university, who are conversant with Participatory Learning & Action (PLA) research, are facilitating this PLA session, which is one among many in a two-year-long research process. In previous meetings, these stakeholders engaged in PLA techniques to assess a range of Guidances and Training Initiatives (G/TIs) related to improving communication between migrants and healthcare professionals. They identified strengths and weaknesses of each G/TI as they perceived them. They exchanged very diverse perspectives and views, learning from and with each other, and co-generated Commentary Charts to record their findings. The atmosphere in this inter-stakeholder group is relaxed, open and trusting, which is essential because the task they face this evening is to use another PLA technique (Direct Ranking) to democratically select a single Guidance or Training Initiative for implementation in a local healthcare setting. First, they review their Commentary Charts and discuss and co-analyse them in relation to the research question asked: ‘Please rank the Guidances and Training Initiatives in terms of ‘most suitable’ to ‘least suitable’ for implementation at local level’. Having listened carefully to all perspectives, they use visual and tangible materials (images, photographs, Post-Its, markers, flipchart paper, paper clips) to complete a Direct Ranking chart which clearly shows their voting result. They check their outcome, and the researchers invite them to confirm their result by engaging in continued discussion, asking key questions such as ‘Is everyone comfortable with the decision you have reached as a group?’ ‘Is there anything of concern to anyone?” Is there anything surprising about your result?’ By the end of the three-hour PLA session, eleven very diverse stakeholders have generated a transparent democratic outcome, based on their co-generation and co-analysis of data. They now know which of the G/TIs they agree to proceed with on their ‘implementation journey’; in future PLA sessions, they will use other PLA techniques to work together, fine-tuning their chosen Guidance for use in the primary care practice where the doctor and practice manager work.

### Evaluation of stakeholders’ experiences of PLA

During Stages 2 and 3 fieldwork, data were generated about stakeholders’ experiences of using PLA techniques to support inter-stakeholder dialogue. A range of data sources provided information for this evaluation of experiences:

- qualitative speed evaluations of the sessions (minuted or audio-taped and transcribed)
- stakeholders at the Irish site completed an in-depth participatory evaluation which provided rich data, augmenting briefer speed evaluations
- researchers at all sites completed extensive post-fieldwork ‘team reflection’ interviews, either face-to-face or by Skype. These included in-depth data from researchers’ perspectives about stakeholders’ experiences of inter-stakeholder dialogue. The team interviews also documented researchers’ reflections on practice as researcher-catalysts (see Table 1) who facilitated inter-stakeholder dialogues. These interviews were audio-taped and transcribed
- research teams completed information-rich fieldwork reports, consistently using the same template, containing qualitative evaluation commentaries. Fieldwork reports and team interviews contained explicit questions designed to evoke rich data about both positive and negative experiences, and potential improvements that might be made. Email correspondence describing researchers’ reflections on transformative moments provided additional data.

In addition, we drew on the following data sources:

- Visual charts, e.g., Commentary Charts, Direct Ranking (all five sites); Seasonal Calendars computerized after fieldwork for analysis and evaluation purposes (all sites but Austria)
- photographic evidence of fieldwork activity and charts (all sites but Austria)
- research team post-fieldwork session debriefing notes which were audio taped and transcribed (Irish site).

### Data analysis of stakeholders’ experiences of PLA

We followed the principles of thematic analysis in qualitative research to analyse our data [70, 74–76]. Two experienced PLA researchers at the Irish site (O’Reilly-de Brun and de Brun) independently generated a ‘start list’ of codes [75, 76] derived from participatory research literature describing a continuum from *positive to negative aspects* of inter-stakeholder dialogue [18, 21, 49, 57]. Collectively, these authors identify positive aspects of stakeholder engagement in terms of *active inclusion* of stakeholders, building *trust and rapport*, supporting *collaboration/collegiality*, promoting *shared and enhanced learning* and *balancing asymmetrical power-relations* among stakeholders. They also identify ‘negatives’ – for example, *exclusion/passivity*, *researcher-controlled process*, and *stakeholder powerlessness*. The ‘start-list’ of codes developed, via repeated readings of researcher and stakeholder data sources, into a final list of thirty-three codes. Each code was understood to incorporate its mirror or binary opposite [77]. Data were coded, then collated to identify emerging themes. Twenty-seven codes containing positive data coalesced into six themes (e.g., collegiality/collaboration); six codes containing negative data coalesced into three themes. (e.g., time commitment). A basic content analysis established the relative weighting of ‘positive’ to ‘negative’ evaluation comments (positive far outweighed negative). The six themes (Table 5) which contained data about stakeholders’ *positive experiences of dialogue*, researchers’ *positive experiences of dialogue* and researchers’ observations and comments about stakeholder’s *positive engagement* offered a measure of richness and triangulation to our analysis and provided the scaffolding for a three-level analysis of what makes for *positive* and *productive* inter-stakeholder dialogue (Fig. 2) [78].

**Table 5:** Three levels of dialogue

Level	Names of Themes A-F
1	A. Trusting relationships in safe space B. Collegiality and collaboration C. Balanced asymmetrical power-relations
2	D. Enhanced learning leading to shifts in understanding E. New relational environments fostering creative problem-solving
3	F. Small leaps of generosity leading to transformative moments/events

## Results

### Positive experiences

Stakeholders reported a wide range of positive experiences of engagement in PLA inter-stakeholder dialogue that were confirmed by researchers’ reports and reflections. The analysis revealed that PLA inter-stakeholder dialogue processes are complex, multi-layered and multi-levelled and unfold in an incremental manner.

Six key themes emerged in three levels of dialogue as shown in Table 5.

Fig. 2 (Flow Chart) provides an overview of fieldwork stages, the PLA techniques used, and how they related to the three levels of inter-stakeholder dialogue identified in Table 5 **.**

Each of the levels is porous and should be understood to have ‘soft boundaries’, meaning that there is interplay between levels, and they are not to be understood as self-contained ‘silos’. For example, key basic components of Level 1, e.g. ‘development of trusting relationships in safe space’ typically flow into Levels 2 and 3. Level 2 (a deepening of Level 1) is unlikely to occur if key components of Level 1 are absent or skipped (e.g., balancing asymmetrical power relations).

#### Level 1 – Basic components of PLA inter-stakeholder dialogue

Stakeholders reported positive experiences of enjoyment and interaction during the early stages of inter-stakeholder dialogue. All findings were relevant across settings, unless stated otherwise below.

The comments below give a flavour of their initial experiences of the PLA dialogic process, highlighting the newness of this approach for them:

*It was a nice and very special experience. I have never done this before in that kind of way. (AUST, SH 01, GP)*

*A new experience and a way of sharing ideas. (GR, SH03, MSU)*

*Very interesting research method. I am very much enjoying the experience, and hoping to learn a lot in the process. (IRL, SH10, MSU/ Int)*

*I love that everyone is putting in the work, there’s a lot of material there and I like also to say thank you because sometimes I feel everyone can be putting their voice in the task, we are all involved, I feel that. It’s good. (IRL, SH11, MSU)*

This positive tone and atmosphere was the foundation for the development of trusting relationships and safe participatory spaces. RESTORE researchers, who, apart from the Irish researchers were all new to this form of dialogic process, reflected on their early experiences as researcher-catalysts facilitating a PLA inter-stakeholder dialogue. English and Austrian teams described achieving genuine engagement with stakeholders and the impact this had on them as stakeholders’ voices began to emerge:

*What I found was how easy it was to get people to engage with the process.* (*ENG, Res)*

*With the group...we have to be respectful and everybody has his or her own voice. It was really... so fascinating - (AUST, Res)*

*It worked and people engaged and there seemed to be enjoyment. And we evolved. (ENG, Res)*

A Dutch researcher was impressed that the PLA process allowed stakeholders from multi-disciplinary backgrounds to collaborate successfully at the early stage of co-designing ground-rules:

*Yeah, what I found remarkable (and I was quite optimistic about the PLA method)... it was a system that people from different multidisciplinary groups were able to get involved in - the whole system, at an early stage already. So they became co-designers a bit ... by this way of working. I think it was very valuable of PLA when you want to implement a strategy or implementation project to do it in this way. I had a very good feeling about it. (NL, Res)*

Greek researchers were surprised to note that high-status medical staff new to this form of engagement became easily involved in it:

*We were also very surprised with how some other medical doctors [got] involved in this PLA process, which was something completely new for the health care staff.* (*GR, Res)*

Researchers noted how PLA techniques operated as vehicles for generating safety in stakeholder groups, and how trusting relationships and positive networking developed through active engagement and teamwork:

*We noticed that the flexible brainstorm technique was a very safe technique for the migrants. It was not too difficult and gave them the opportunity to associate and discuss with each other in a natural way. We had the impression that this technique facilitated the contact between the migrants and the researchers. What was interesting was that SH16 felt safe enough to tell to the other migrant and researchers that he was undocumented. (NL, Res)*

*Each PLA technique achieved what we hoped for; every exercise produced lots of information and was very interactive, the techniques assisted in untangling issues by talking through them and breaking down ideas into tasks. Above all, it is still achieving… engagement of the stakeholders. (GR, Res)*

At the English and Dutch sites, which involved very diverse stakeholder groups, researchers noted how rewarding inter-stakeholder dialogue appeared to be – how ‘safe space’ made it possible for stakeholders to begin to offer their perspectives in a collegial and collaborative manner; this helped to balance asymmetrical power-relations in a new group where some stakeholders possessed more social capital and/or professional power than others:

*As researchers we have found it very rewarding working with the group, who all appear to get on well and have been willing and able to work in a democratic and inclusive manner, respectfully listening to each other and bringing an impressive degree of knowledge and expertise to the stakeholder table. It has been encouraging witnessing a great level of networking taking place within the group, which suggests that stakeholders are benefiting from the meetings in ways that go beyond the RESTORE project. (ENG, Res)*

*Additionally, we [researchers] noticed that the mixed groups really stimulated interaction of all ‘sorts’ of staff members, we saw in depth [PLA focus group] discussions in which all group members were participating. (NL, Res)*

#### Level 2 – Deeper engagement: Additional necessary components of a PLA inter-stakeholder dialogue

Level 2 dialogue was a continuation and ‘deepening’ of the trust and safety developed at Level 1. As described previously, the practical tasks for stakeholders during Stage 2 fieldwork were to review and assess G/TIs relevant to their primary care setting, using PLA techniques (Commentary Charts and Direct Ranking, as per Table 3) to select a single G or TI for actual implementation. Stage 3 fieldwork involved fine-tuning, planning and implementation activities (using Seasonal Calendars and Flexible Brainstorming techniques, among others). The comments below illustrate the key necessary components (see Fig. 2) of Level 2 inter-stakeholder dialogue – sharing of diverse perspectives, which led to enhanced learning, which fostered shifts in understanding:

*I really liked the fact of being heard among those people – my voice had the same weight as all the others*. (*AUS ,SH07, MSU)*

*The participatory approach was very interesting as well as how we exchanged ideas and knowledge and especially the cohesion of the group.* (*GR, SH10)*

*Very democratic and led to better understanding of the G/TIs and other stakeholder’s views and input. ( IRL, SH01, GP)*

Researchers echoed this Level 2 engagement in their reflective comments about stakeholders’ continued active inclusion and the balancing of asymmetrical power-relations:

*And they [stakeholders] were on different levels, I mean there was a doctor, a GP and another one, a local administrator, so they would have differences... Yes, but they were accepting, the one was accepting the other. (GR, Int)*

An exchange between Dutch researchers during a post-fieldwork team reflection interview highlights their concern and relief about balancing asymmetrical power relations:

*Yes, it worked, we were a little afraid that working in a multidisciplinary setting with different groups...[might not work] ( NL, Res 1)*

*Certainly in groups with hierarchical differences.... (NL, Res 2)*

*Yes, which would be difficult... there would be a group dynamic... the participants with the highest educational levels would dominate the discussion or say, okay, I’m not saying anything because I want to give... migrants more opportunity to say something. But it worked quite naturally and that was surprising. (NL, Res1)*

At Level 2, ‘shifts in understanding’ occurred, even though this involved ‘hard work’ – this was articulated in particular by the stakeholders in the Irish setting:

*I find this experience very enjoyable. Because we are doing some work here, it’s hard work but it’s very, very enjoyable and I think it really broadens your perspective on things, and new horizons. When I reflect back on the first meeting we had, I remember when I was reading the first, em, Guidance I could only see it from my own perspective as an interpreter, and now, when I was reading it today, I could really, I started seeing it from other perspectives and I think that shows that we’re really learning things for ourselves as well, so it’s great.* (*IRL, SH07, Int)*

The incremental nature of the inter-stakeholder dialogue, the co-operative relational environment and enhanced learning enabled stakeholders to shift to new positions or understandings:

*It’s been team work, people from different backgrounds coming together for a bigger purpose but actually having a great time doing it* ***.*** *I think the trust and openness and everything has been phenomenal and I learnt so much along the way. I would have my own fixed ideas, you know, about consultations and interpreted consultations, and the good/bad and indifferences associated with that. But to have it cracked wide open every time and hearing all the different perspectives, I think, in particular, from the service users, but also from the community interpreters, and to see the different slants – I learnt so much about the interpreting [process] that I would never have known.*

*(IRL, SH01, GP)*

*It’s a marvellous way to learn...You [indicating other stakeholders] have changed my mind on subjects that I thought I was entrenched in, that I had an entrenched view on.*

*(IRL, SH05, PM)*

Another component of Level 2 inter-stakeholder dialogue is creative problem-solving. This tended to occur because a new relational environment (based on co-operation and collaboration rather than competition) generated co-operative discussion which stakeholders linked to the ability to creatively solve problems together:

*The only truly group discussion I’ve ever been part of – a properly democratic process that actually solves problems.* (*IRL, SH02, PM)*

Level 3 – Optimal components of a PLA inter-stakeholder dialogue: Potential for ‘transformative moments’

The context and pre-condition for Level 3 dialogue (see Fig. 2) is when stakeholders encounter a seemingly intractable problem. This may or may not happen during a PLA dialogue, but should an intractable problem surface, the groundwork laid during Levels 1 and 2 dialogue tends to create the necessary environment in which stakeholders may reach across boundaries (e.g., organisational, lay/professional, status/power) to generate a creative solution to the problem. This outcome is what we coin a ‘transformative moment’ or event. It is important to emphasise that such an event is not a *required* or *necessary* component of PLA inter-stakeholder dialogues, which are successful and complete in themselves without this, therefore we do not expect a transformative event to occur in all PLA dialogues. In RESTORE, three countries reported experiences of transformative moments (England, Ireland, The Netherlands.) The Irish example below illustrates how Level 3 inter-stakeholder dialogue can carry forward all that is experienced and achieved in Levels 1 and 2, providing a platform for Level 3 to unfold.

While collaborating on their Seasonal Calendar, Irish stakeholders noticed an emerging problem: the practice staff had assigned themselves a large number of key implementation tasks which needed to be completed at the outset of the implementation of the G/TI. This left them with a very daunting and overwhelming workload early on in their implementation journey. Irish researchers’ post-PLA session team reflection captured a sense of the burden this generated for practice staff stakeholders:

*I think our GP staff tonight felt heavily burdened... they probably felt a fair responsibility that they’ve a lot to do. One of them kept saying: ‘There’s all that stuff [practice staff tasks] there on the top line - that should have been done yesterday, and there’s a lot there...’ (IRL, Res)*

Practice staff took on the tasks in the belief that they were the only stakeholders who could complete them. However, other stakeholders noticed the dispiriting effect of this burden on the practice staff. A lively and compassionate conversation ensued: community interpreters, migrant service-users and the policy planner offered to lift some of the workload burden from the shoulders of practice staff. They negotiated and re-positioned tasks on the Calendar, redistributing them more evenly across all stakeholders. This brought a great sense of relief to practice staff:

*Yeah, it seems a bit more in control now, I was worried about that top line [of practice staff tasks]* *so much to do!* *Once we’ve broken it down into what we need to do now, and when, and who’s going to help us [to] do it, it’s cool, grand, I'm good. (IRL, SH02, PM)*

*Likewise – yeah, I think I’ve also been a bit concerned about the fact that so much of it now would revolve around us [practice staff] trying to recruit people, and how are we going to do this, oh god, will we do it wrong? I think it’s become obvious that it’s very much shared and we have help from all the excellent resources, be it from the service-users or interpreters - there’s a lot of input there, which means it won’t be all down to us. (IRL, SH01, GP)*

This example of a ‘transformative moment’ for stakeholders highlighted the impact of the deepening of relationships of trust. The increasingly co-operative and collaborative nature of the PLA inter-stakeholder dialogue and engagement made it possible for some stakeholders to take a *small leap of generosity* towards others. This directly and effectively resolved the potentially intractable problem. It allowed the inter-stakeholder group to resolve this potentially significant barrier to continued implementation.

Post-PLA session debriefing notes by the Irish researchers confirmed this:

*...it became very clear that the community interpreters, while they had highlighted only three [tasks for themselves] were quite willing to get involved in [the] pink ones [tasks], which were the practice-assigned ones. And I could visibly see... a relaxation coming over the practice staff. Because they were hearing, unambiguously, from the community interpreters that: ‘No, we are very willing to collaborate and work with you to work [this] out.’ The service-users, policy person, and community interpreters were... generously saying: ‘Don’t worry, you are not on your own; we’ll help you, we’ll translate the posters, we’ll look at the names, we’ll help you to identify service-users with these languages’, etc, etc. I could see that happening, I could see that moment when things began to not look so frightening and so burdensome for the practice staff. They are the locus of so much of what happens in [this part of] the project. It’s a tough one for them in that way*. (*IRL, Res)*

### Negative experiences

In contrast to the ‘positives’ noted above, negative experiences reported coalesced into three key analytical themes: *time commitment*, *research fatigue* and *uptake of implemented service*. GP and clinical stakeholders reported experiencing time challenges because of the commitment involved in a lengthy and intensive research engagement:

*But I still do think as a GP it’s hard to adjust to spending so much time on this, but very worthwhile obviously. As always, I think the project is fascinating.’ (IRL, SH1, GP)*

Researchers had time challenges related to fitting in all the planned PLA processes in fieldwork sessions, while at the same time not over-burdening stakeholders, and also in terms of having insufficient time to reflect on fieldwork:

*We have noticed that we always run short of time at our sessions. For example at our 2* ^*nd*^ *[Stage 3 fieldwork] session we had planned to start the seasonal calendar and we did not have time for this, and in our 3* ^*rd*^ *session the discussion was flowing so well and again we did not start the seasonal calendar. We worked around the schedule always and found other ways and time to complete the PLA techniques. (GR, Res)*

*A major inhibiting factor often mentioned is the fact that the GP practice is always in shortage of time. As research team, we really have to be careful not to overload the practice with too many questions or tasks. (NL, Res)*

*it has been very challenging to protect sufficient time to fully reflect on and analyse the session transcripts which emerge from [fieldwork] sessions... there is a constant tension and an art to finding the balance: we sense the need to slow down the research process to ensure that, as the field researchers, we don’t overlook and ignore important information from stakeholders, thereby missing opportunities to generate solutions to genuine problems of local adaptation, and concomitantly compromising our PLA values of respect for stakeholders’ expertise and experience. On the other hand, we know how important it is to maintain momentum, so that stakeholders feel they are advancing and progressing. (IRL, Res)*

While rare, there were reported instances of research fatigue on the part of both researchers and stakeholders:

*As researchers, we too can burn out and find our energy for the project compromised as meetings build up in both intensity and number... The Irish stakeholder group can have up to 12 lively vocal people engaging all at once and can be demanding in terms of facilitation, group management and time management skills. (IRL, Res)*

*Another challenge… was the heavy workload that the stakeholders had these past few months. We felt that we were becoming quite exhausting [for them]. They did not express this towards us, but at times we felt that they just need a break! They appreciated that we did not bother them during their August holiday and they came back rejuvenated, and that was great. (GR, Res)*

In two countries (Ireland and Greece), as a result of the RESTORE implementation journey in local settings, a new interpreting service was introduced into primary care practices but uptake was low. This contributed to a sense of research fatigue and frustration on the part of stakeholders and researchers alike:

*…We feel that the stakeholders are burned out in general with their position and extra work loads they have now [in their work-lives] and they are bummed [very disappointed] that this implementation isn’t going as well as they thought. Likewise for the researchers, we are surprised that it is going so ‘slow.’ (GR Res)*

## Discussion

### Summary of findings

Our findings show that using a PLA mode of engagement and techniques for inter-stakeholder dialogue in a recent European primary healthcare implementation project led to a meaningful engagement of stakeholders who came from diverse backgrounds with significant power asymmetries. It provided opportunities for stakeholders to share, in an open and democratic manner, their diverse expert ‘knowledges’ about the suitability or otherwise of guidelines and training initiatives. Thisallowed them to select one for implementation and to contribute in practical terms to the implementation process at a local level. Stakeholders reported predominantly positive, but also a few negative experiences of inter-stakeholder dialogue. This was true across stakeholder groups in all study centres. Researchers’ fieldwork reports and interviews confirmed these results. We have presented the elements contributing to a positive and productive inter-stakeholder dialogue in terms of three levels of dialogue which develop incrementally. The development of trusting relationships in safe space fosters collegiality and co-operation (Level 1) moving towards enhanced learning across stakeholder groups/interests. This can lead to shifts in understanding and generates a relational environment of collaboration (Level 2) which may lead to experiences of compassionate co-responsibility in the face of seemingly intractable problems (Level 3). In such instances, the key experience is one of burden-sharing, prompting a ‘small leap of generosity’ from some stakeholders towards others which resolves the impasse or problem. The outcome is what we have coined a ‘transformative moment’ for the stakeholders involved. This can be a pivotal moment in an implementation journey – where there is a danger that an intractable problem might threaten progress, but a small leap of generosity re-energises stakeholders and impels them, and the research, forward.

Negative experiences were relatively few and related to time commitment, research fatigue and disappointment regarding lack of uptake of implemented services.

### Findings discussed in relation to the literature

There is limited knowledge about suitable methods for involving stakeholders in a meaningful (rather than tokenistic) inter-stakeholder dialogue [15, 17–30].

Tierney et al. note that participatory approaches and methods seem promising and call for further research and methodological innovation [30, 65, 74]. In line with this, our findings show that, in RESTORE, the use of a PLA ‘mode of engagement’ and series of PLA techniques for inter-stakeholder dialogue about implementation went beyond what Beresford describes as a ‘consumerist approach’ and proved effective as a democratic method of facilitating positive and productive dialogue among diverse stakeholders in diverse primary care systems.

The empirical data show that PLA provided a ‘safe space’ and promoted trusting relationships [58] in which stakeholders felt sufficiently secure to share their diverse perspectives and opinions. Researchers, as a function of their training in PLA, had skills to actively encourage stakeholders to recognise that they are ‘experts’ in their own right and encouraged them to bring their unique *implicit* knowledge to the stakeholder table, making it explicit to the research process through dialogue [19, 22, 32, 60].

There were also some differences across stakeholder groups: the challenges of clinician engagement in primary care research in general, in particular related to the time involvement, is well documented [79] and our experience was similar in that regard. However, we acknowledge an important distinction - clinician’s involvement in PLA fieldwork in RESTORE was longer than in studies using more traditional methods, e.g., interviews or focus groups, and was sought over an extended period of time (20 months for fieldwork). Interestingly, we observed that clinicians who were engaged gave more time than they thought they could, and were willing to explore creative ways of being involved in a time efficient way (we described this in more detail in Lionis et al. [59] and Teunissen et al.) [69]. Negative experiences related to policy circumstances that could not be influenced by the stakeholders are similarly reflected in the literature [80].

### Methodological critique and suggestions for future research

We have documented and discussed key methodological strengths of our study above. Here, we note some areas for improvement and make some suggestions about how future research studies could be strengthened.

#### Limitations of our study

1. We were unable, for site-specific ethical reasons, to include the use of stakeholder evaluation comments from the English site in our thematic analysis. However, we were able to ameliorate this by including data from researcher’s reflection interviews and fieldwork reports from this site.
2. Our thematic analysis about the use of PLA was conducted after the RESTORE project ended. This precluded us from incorporating stakeholders’ evaluation criteria into the analysis, as we would prefer and recommend for all PLA projects. However, the numerous and detailed stakeholder verbatim quotes included by researchers in their fieldwork reports, and researchers’ own insights offered during post-fieldwork reflection interviews, provided valuable pointers for identifying appropriate evaluation criteria, analytical codes and themes and supported interpretation of findings.
3. Evaluation methods: ‘speed evaluations’ are time-efficient but brief. A practical way of eliciting immediate responses from stakeholders at the close of PLA sessions, they allow researchers and stakeholders to hear how an inter-stakeholder dialogue is developing, what is working well and what requires improvement. Improvements can then be worked into the next PLA session. However, speed evaluations should ideally be augmented by participatory evaluations (as was the case in the Irish site) to give stakeholders adequate time and opportunity to engage in more in-depth evaluation, and to collaboratively include criteria stakeholders consider appropriate and valuable. This often fills ‘evaluation gaps’ the researchers are unaware of, and generates unanticipated results which are highly beneficial to the progress of the research.
4. In common with all qualitative studies, we do not claim generalizability of findings for the study data presented here but suggest that our results about using a PLA ‘mode of engagement’ and PLA techniques to meaningfully engage stakeholders in inter-stakeholder dialogue across five European countries with very different primary care systems are promising and that PLA may be transferrable to other countries, topic areas and community or population groups.

#### Recommendation for future research

To add to the evidence base, we need further research and evaluation to explore if and how PLA techniques might work when applied in projects with very different research foci and stakeholder groups than those in RESTORE.

Two of the three negative themes identified (time commitment, workload/research fatigue) are important issues for careful consideration in future PLA projects. In RESTORE, we provided clear information at the outset, on-going reflection on the process and support for the workload. Nevertheless, the time commitment, as it evolved, became challenging for both clinical stakeholders and researchers. However, we have noted that, overall,those involved thought the time commitment was worth it. For future PLA projects, wherever funding, bureaucratic and/or other circumstances allow, we recommend extensive collaborative discussion between representative stakeholders and researchers at the *very earliest stages of project design* to develop and discuss ideas for managing time, acknowledging ‘upfront’ that the organic and iterative nature of a PLA project may affect this, and agreeing (as we did in RESTORE) that alterations in timeframe will be negotiated and re-negotiated where possible.

The critical importance of the researchers as catalysts and facilitators of participatory behaviour in research settings is clear. This was skilled work and the researchers were trained by two of their group who already had the skills and knowledge. Further, there was a continual learning feedback loop into the researcher group from the fieldwork as to application of the methodology. A key question, therefore, is how participatory learning and action can be replicated without those skills and without the funding to support their purchase? Arguably this question is about building capacity in the academic primary care community for PLA research. Also, it is about advising that PLA research is not undertaken by those without the necessary skills set, no more than a randomized controlled trial would be conducted without the required skill set.

## Conclusions

PLA, with its mode of engagement and range of techniques, is capable of involving stakeholders in a meaningful, positive and productive inter-stakeholder dialogue across diverse primary health care systems. PLA attends to power differentials within and between stakeholder groups, making it particularly effective for facilitating equity between differing and conflicting perspectives as they emerge in inter-stakeholder dialogues. This makes PLA a valuable approach to be used in the further development of community-based primary healthcare.

PLA inter-stakeholder dialogue processes are complex. Our analysis describes three levels of dialogue which develop incrementally. This analytical framework may prove useful to researchers interested in developing and supporting meaningful stakeholder engagement through inter-stakeholder dialogues in primary healthcare research.

## Abbreviations

CBPR: Community based participatory research
GP: General practitioner
GUIDELINES AND TRAINING INITIATIVE: Guideline / training initiative
Int: Interpreter
MSU: Migrant service user
NPT: Normalisation process theory
PAR: Participatory action research
PLA: Participatory learning and action
PM: Practice manager
PN: Practice nurse
PO: Practice operator
PPI: Public and patient involvement
PR: Participatory research
PRA: Participatory rural appraisal
Res: Researcher
SH: Stakeholder
